# Stroke in a Patient With Pulmonary Siderosis: An Unusual Association

**DOI:** 10.7759/cureus.68360

**Published:** 2024-09-01

**Authors:** Jagannath S Dhadwad, Kunal Modi, Janvi Panchal, Prince R Yadav, Chandan Dash

**Affiliations:** 1 Internal Medicine, Dr. D.Y. Patil Medical College, Hospital and Research Centre, Pune, IND; 2 General Internal Medicine, Dr. D.Y. Patil Medical College, Hospital and Research Centre, Pune, IND; 3 General Medicine, Dr. D.Y. Patil Medical College, Hospital and Research Centre, Pune, IND

**Keywords:** iron ore, occupational lung disease, interstitial lung disease, stroke, pulmonary siderosis

## Abstract

Occupational lung diseases are a major hazard, which can lead to severe complications and a worsening quality of life. Out of these diseases, pulmonary siderosis was considered an innocuous disease. We detail the case of a 42-year-old man who had a history of chronic exposure to ferrous fumes due to his occupation. He presented with breathlessness and symptoms of a stroke. CT imaging studies showed an ischemic infarct in the brain and changes suggestive of interstitial lung disease in the chest, which was eventually diagnosed as pulmonary siderosis. In spite of having no comorbidities and significant past history, the patient developed a debilitating condition most likely as a consequence of the underlying lung pathology. We want to highlight the importance of early detection and proper management of interstitial lung diseases.

## Introduction

Pulmonary siderosis is an uncommon occupational lung disease, seen in about 7% of welders [1]. It develops due to prolonged inhalation of fine ferrous particles. Due to the absence of substantial signs and symptoms, as well as accompanying fibrosis, it was formerly thought to be a benign pneumoconiosis [2,3]. There have been reports of symptomatic disease associated with it, even in conjunction with interstitial fibrosis [3-5]. There is an increased risk of developing pulmonary siderosis in mining, welding, iron oxide, iron ore, and other manufacturing and jewelry industries [3,5,6].

Initially thought to be a benign disease that does not evoke much of a reaction, recent reports have suggested that pulmonary siderosis may be associated with some degree of fibrotic changes. These cases usually present with symptoms such as breathlessness and persistent cough. Long-term exposure, or in some cases, brief exposures with high intensity may lead to respiratory complications like chronic bronchitis and a decline in pulmonary function [7]. However, cardiovascular and cerebrovascular events secondary to pulmonary siderosis have not been observed in adults.

Hence, we try to demonstrate the diagnostic difficulties associated with a case of stroke in a patient with pulmonary siderosis.

## Case presentation

We present the case of a 42-year-old male, an iron ore worker by occupation. He presented with weakness of the right upper and lower limbs for two days associated with slurring of speech and breathlessness for the past month, which had aggravated in the past one day. The weakness of the limbs was non-progressive in nature and was not associated with facial deviation. This, however, was associated with dyspnea, MMRC (Modified Medical Research Council) II initially, and had since worsened to grade IV. A working diagnosis of acute stroke was made with breathlessness under evaluation.

He had no history of diabetes mellitus, hypertension, ischemic heart disease, or any other comorbidities. He gave no history of smoking or tobacco chewing, nor any prior trauma/surgeries. There was no history of early cardiovascular deaths in the family.

On examination, his blood pressure was normal at 130/80 mmHg, and his pulse rate was 98 beats per minute. There was no pallor, icterus, cyanosis, clubbing, or lymphadenopathy seen. The patient was tachypneic on rest with a respiratory rate of 30 per minute and a room air saturation of 81%. The patient was initiated on non-invasive ventilation and evaluated further. On auscultation of the respiratory system, bilateral vesicular breath sounds with bilateral fine crepitations over all the lung fields were noted. The neurological assessment revealed an increased tone and reduced power in the right upper and lower limbs (1/5), with Babinski positive on the right and normal findings on the left. There was no obvious murmur noted on cardiovascular system auscultation nor was there a carotid bruit.

His arterial blood gas (ABG) was noted to have high PaCO_2_ and low PO_2_ suggestive of type two respiratory failure (Table 1). He was intubated, as there was breathlessness and progressively worsening mentation (Glasgow Coma Scale score: 12/15 to 7/15)

**Table 1:** ABG ABG, arterial blood gas

Parameter	Value	Reference range
pH	7.29	7.35-7.45
pCO_2_	64 mm of Hg	35-45 mm of Hg
Bicarbonate (HCO_3_)	27 mEq/L	21-28 mEq/L

**Figure 1:**
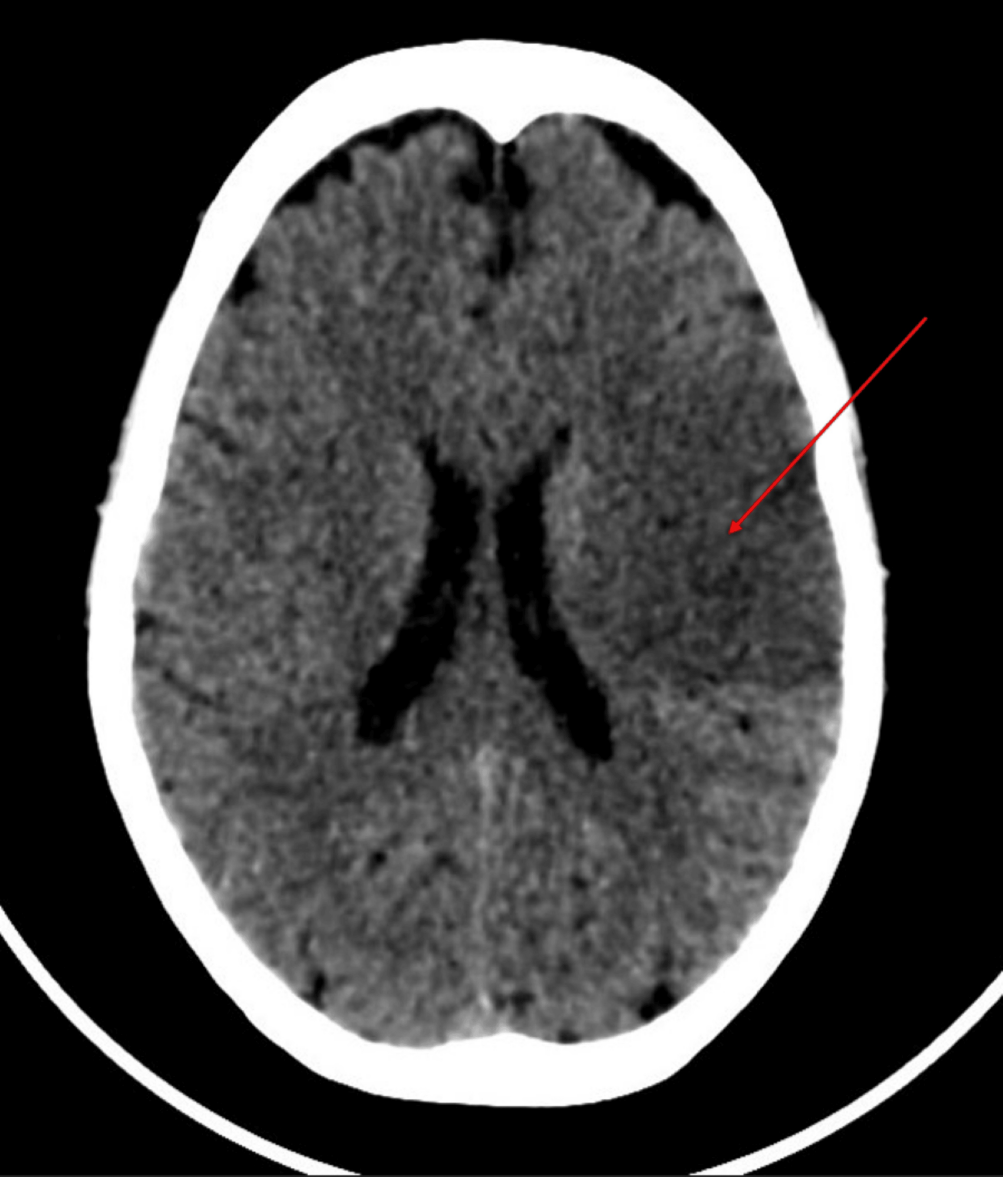
CT scan of the brain showing acute non-hemorrhagic infarct in the left fronto-parietal region (red arrow)

A CT brain was performed, which revealed an acute non-hemorrhagic infarct in the left fronto-parietal region (Figure 1).

**Figure 2:**
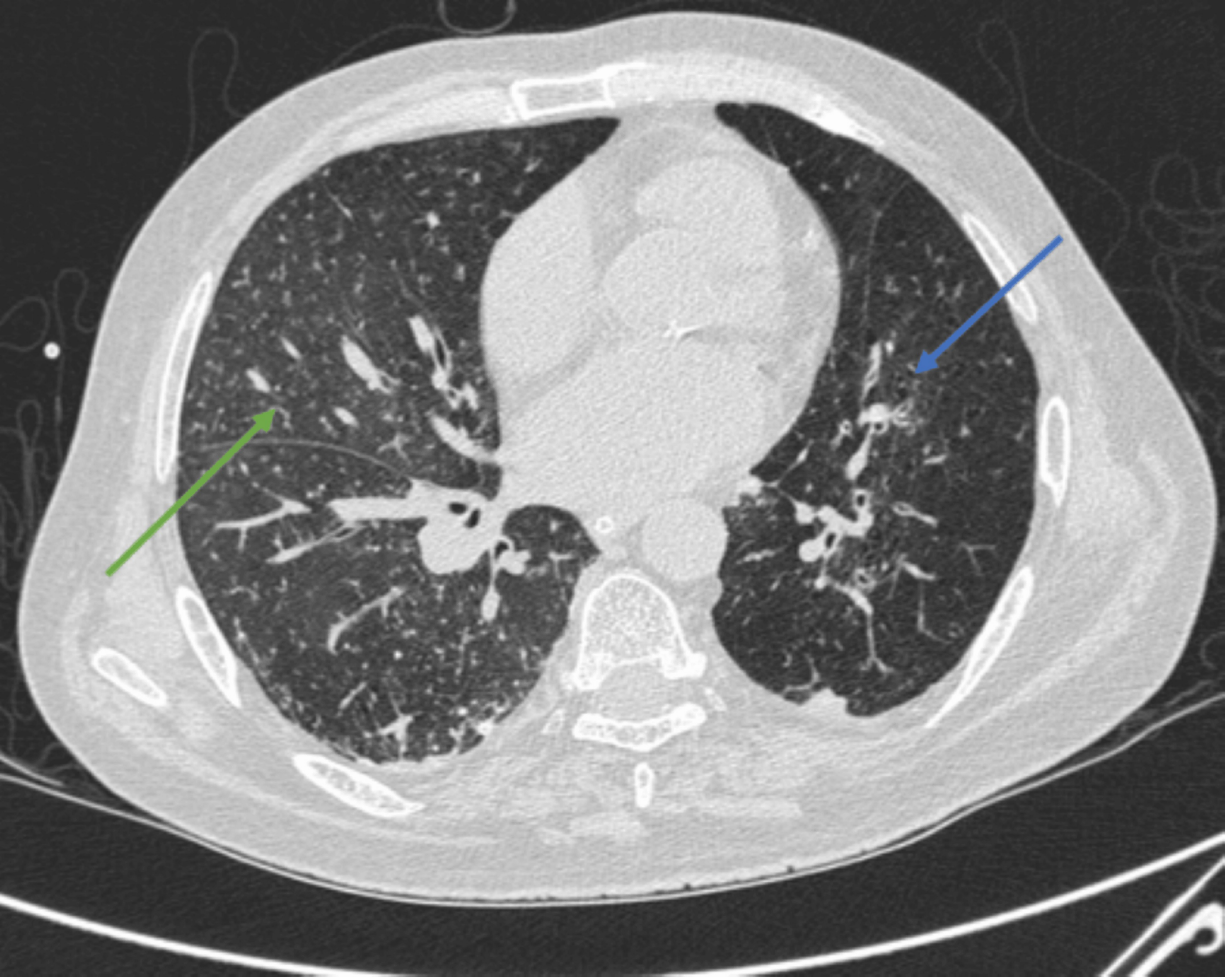
CT scan of the chest showing centrilobular emphysema (blue arrow) and mosaic attenuation with centrilobular nodules (green arrow) suggestive of interstitial disease

**Figure 3:**
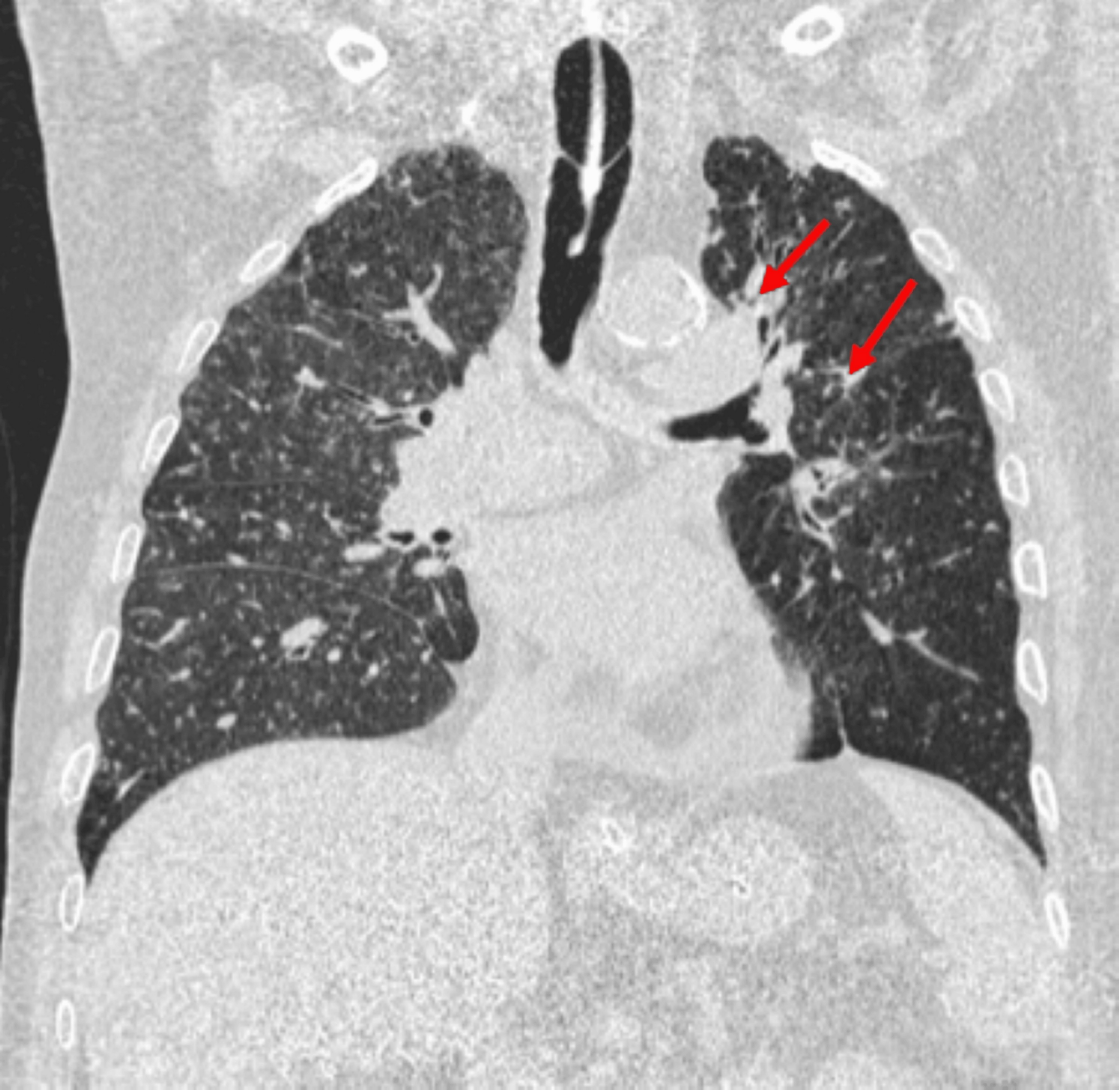
CT scan of the chest showing fibronodular changes (red arrow)

To further evaluate the respiratory compromise, a CT scan of the thorax was done, which showed diffuse centrilobular nodules in bilateral lungs and fibronodular changes in apical segments of both lungs, with centrilobular emphysema and subpleural hyperdense consolidation, suggestive of interstitial lung disease - most likely pulmonary siderosis in view of his occupation (Figures 2, 3).

Blood investigations revealed elevated inflammatory markers, C-reactive protein (CRP), and erythrocyte sedimentation rate (ESR), with remaining parameters well within normal limits for his age (Table 2).

**Table 2:** Blood parameters SGOT, serum glutamic oxaloacetic transaminase; SGPT, serum glutamate pyruvate transaminase; ALP, alkaline phosphatase; Na, sodium; K, potassium; Ca, calcium; Mg, magnesium; T3, triiodothyronine; T4, thyroxine; TSH, thyroid stimulating hormone; HbA1c, glycated hemoglobin; HDL, high density lipoprotein; LDL, low density lipoprotein; CRP, C-reactive protein; ESR, erythrocyte sedimentation rate

Parameter	Observed value	Reference range
Hemoglobin	14.2 g/dL	13.2-16.6 g/dL
Total leukocyte count	7800/µL	4000-10000/µL
Platelet	151000/µL	150000-410000/µL
Bilirubin (total)	0.74 mg/dL	0.22-1.20 mg/dL
Bilirubin (conjugated)	0.27 mg/dL	≤0.5 mg/dL
Bilirubin (unconjugated)	0.47 mg/dL	0.1-1 mg/dL
SGOT	34 U/L	8-48 U/L
SGPT	37 U/L	7-55 U/L
ALP	64 U/L	40-129 U/L
Protein (total)	7.4 g/dL	6.4-8.3 g/dL
Albumin	4.6 g/dL	3.5-5.2 g/dL
Na^+^	145 mmol/L	136-145 mmol/L
K^+^	3.86 mmol/L	3.5-5.1 mmol/L
Urea	18 mg/dL	17-49 mg/dL
Creatinine	1.15 mg/dL	0.6-1.35 mg/dL
Ca^2+^	8.7 mg/dL	8.6-10.2 mg/dL
Mg^2+^	1.8 mg/dL	1.8-2.4 mg/dL
T3	0.66 ng/mL	0.64-1.52 ng/mL
T4	8.68 µg/dL	4.87-11.72 µg/dL
TSH (ultrasensitive)	0.50 µIU/mL	0.35-4.94 µIU/mL
HbA1C	5.4%	4-5.6%
Cholesterol (total)	125 mg/dL	<200 mg/dL
Triglycerides	110 mg/dL	<150 mg/dL
HDL	27 mg/dL	>40 mg/dL
LDL	86 mg/dL	<100 mg/dL
CRP	172.26 mg/L	<2 mg/L
ESR	26 mm/hr	≤20 mm/hr
Homocysteine	11 µmoles/L	5-15 µmoles/L

**Figure 4:**
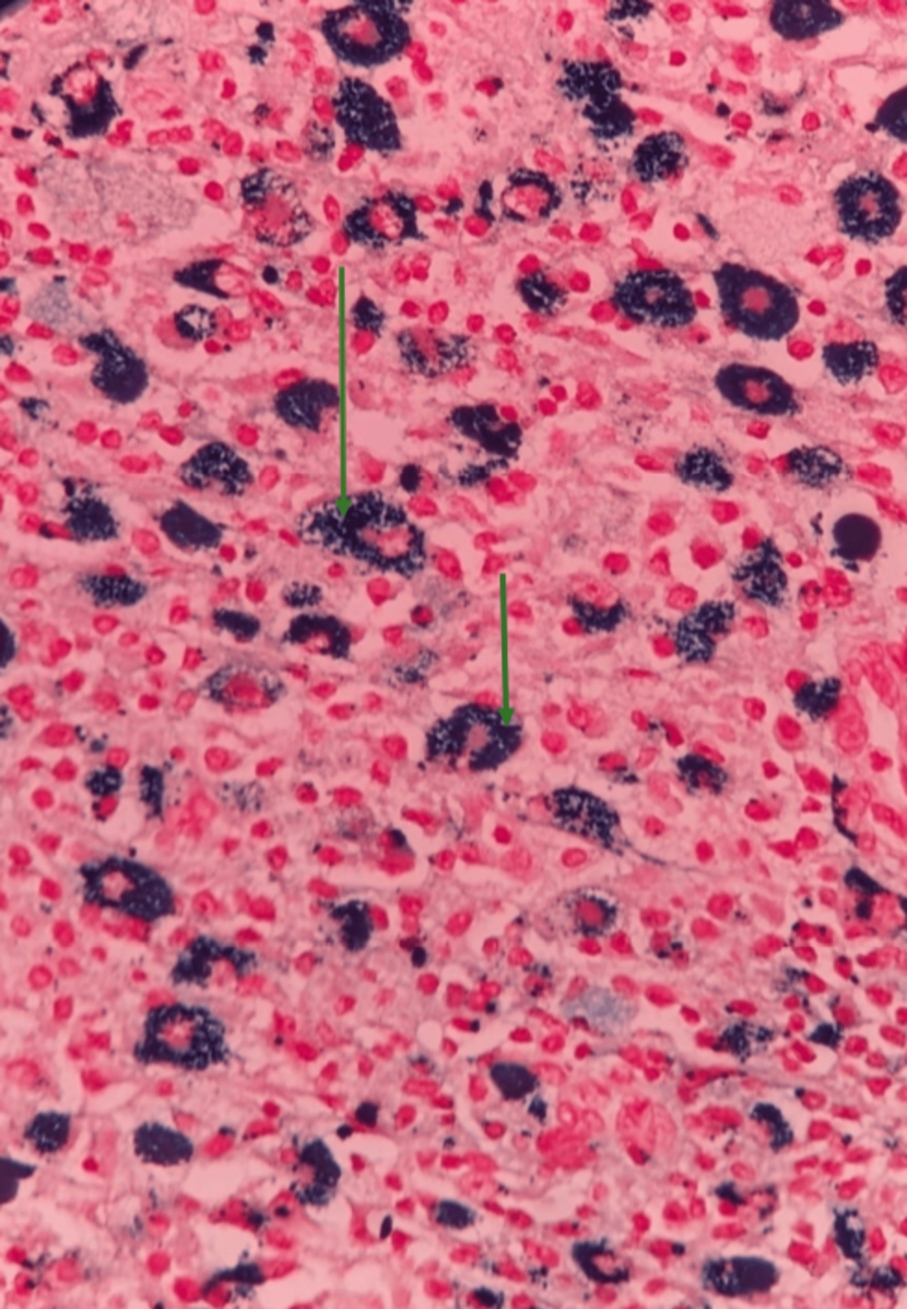
Biopsy (100x magnification) showing macrophages with iron deposition, positive PERL stain (green arrow)

His 2D echo showed a normal study with an ejection fraction of 60% and no regional wall motion abnormality. Antiphospholipid antibodies (APLA) and antineutrophil cytoplasmic antibodies (ANCA) were not detected. A bronchoscopy-guided biopsy was done, which showed positive PERL staining, confirming the diagnosis of pulmonary siderosis (Figure 4).

Based on all the investigations done, a final diagnosis of acute stroke with pulmonary siderosis was made. The patient was started on dual antiplatelet therapy along with symptomatic management for breathlessness that included nebulization. Intravenous steroids were given for acute exacerbation. Rehabilitation therapy including physiotherapy was started for the limb weakness. He was eventually weaned off the ventilator but required intermittent BiPAP support. The patient was finally discharged with the advice to continue home BiPAP and physiotherapy.

## Discussion

Worker exposure to metal vapors containing iron in various industries is the most common cause of pulmonary siderosis. McLaughlin originally published the first case series of siderosis in welders in the year 1936. He followed 15 of the cases for a period of nine years. He demonstrated that there was no long-term severe radiological progression or severe lung functional impairment. There was no evidence of lung fibrosis after 40 years of iron oxide exposure [3].

Another study conducted by Meyer E. and an associate showed no fibrotic changes in the histopathological examination of the lungs of patients who were exposed to iron fumes [8].

A majority of previous studies and reports suggest that pulmonary siderosis rarely causes progressive respiratory or other systemic complications. Contrary to these, we highlight this case in which the patient had extensive pulmonary involvement and presented with a cerebrovascular accident with no other known risk factors.

Studies have shown that interstitial lung diseases evoke a pro-inflammatory state in the body with an increase in various interleukins and cytokines. Alveolar macrophages are stated to play an important role. These macrophages increase in number to ingest the inhaled particles and release inflammatory markers, cytokines, and oxidants in the activation stage. The lung fibroblasts and epithelial cells are also theorized to release secondary markers. This could be a possible explanation for the cerebrovascular event in this patient [9-11].

As a consequence of the seemingly inert nature of iron compounds, hemosiderosis of the lung is considered to be a benign form of pneumoconiosis. Despite its inert nature, it can lead to complications related to iron deposits in the lung causing fibrosis or because of distal spread. In this case, the presentation was unusual with hemiparesis, which then revealed the underlying pulmonary siderosis.

Within the scope of this particular case, the HRCT chest scan revealed several nodular opacities together with reticulation. These findings described above, however, do not constitute confirmation of the diagnosis. The histopathological investigation is essential for the definitive diagnosis. In the present study, the imaging was contributory in view of his occupation, and the diagnosis was confirmed by biopsy.

## Conclusions

Stroke is a rare complication of occupational pulmonary diseases, and its pathophysiology is not clear. More complicated pulmonary siderosis cases are being reported, which should prompt research toward its association with other systemic complications. To the best of our knowledge, this is a rare case of stroke following occupational iron exposure. Management of the underlying lung parenchymal lesion with timely diagnosis can help reduce the associated neurological impairment.
